# Fatal gastrointestinal toxicity with ipilimumab after BRAF/MEK inhibitor combination in a melanoma patient achieving pathological complete response

**DOI:** 10.18632/oncotarget.10651

**Published:** 2016-07-18

**Authors:** Maria Gonzalez-Cao, Aram Boada, Cristina Teixidó, María Teresa Fernandez-Figueras, Clara Mayo, Francesc Tresserra, Jean Bustamante, Santiago Viteri, Enrique Puertas, Mariacarmela Santarpia, Aldo Riso, Feliciano Barron, Niki Karachaliou, Rafael Rosell

**Affiliations:** ^1^ Translational Cancer Research Unit, Instituto Oncológico Dr Rosell, Dexeus University Hospital-Quirónsalud Group, Barcelona, Spain; ^2^ Dermatology Department, Hospital Universitari Germans Trias i Pujol, Badalona, Barcelona, Spain; ^3^ Pathology Department, Hospital Universitari Germans Trias i Pujol, Badalona, Spain; ^4^ Pathology Department, Dexeus University Hospital-Quirónsalud Group, Barcelona, Spain; ^5^ Albert Einstein Medical Center, Philadelphia, PA, USA; ^6^ Radiotherapy Department, Hospital Quirónsalud, Barcelona, Spain; ^7^ Medical Oncology Unit, Human Pathology Department, University of Messina, Messina, Italy; ^8^ Medical Oncology Unit, Insituto Nacional de Cancerología, México; ^9^ Catalan Institute of Oncology, Cancer Biology & Precision Medicine Programme, Germans Trias i Pujol Hospital and Health Sciences Institute, Badalona, Spain; ^10^ Pangaea Biotech, Laboratory of Oncology, Barcelona, Spain

**Keywords:** BRAF mutation, ipilimumab, melanoma, sequential treatment, toxicity

## Abstract

Approximately 50% of metastatic melanoma patients harbor BRAF mutations. Several treatment options including the combination of BRAF and MEK inhibitors (BRAF/MEKi) and immunotherapy (mainly anti CTLA-4 and anti PD-1 antibodies), have been shown to improve survival in these patients. Although preclinical data support the synergistic effect of both modalities in combination, data confirming the activity and tolerability of these combinations are not yet available in the clinical setting. Herein, we report the case of a melanoma patient treated with sequential BRAF/MEKi (dabrafenib plus trametinib) followed by the anti CTLA-4 antibody ipilimumab who achieved a pathological complete response. Unfortunately, the patient died due to fatal gastrointestinal (GI) toxicity. Analysis of the BRAFV600E mutation in circulating tumoral DNA (ctDNA) from peripheral blood samples and serial tumor tissue biopsies throughout treatment demonstrated a good correlation with clinical evolution.

## INTRODUCTION

In recent years, several drugs have been approved for the treatment of patients with advanced stage melanoma harboring BRAF mutations. Two main treatment strategies have been shown to improve survival: the combination of targeted inhibitors of BRAF (such as dabrafenib or vemurafenib) and MEK (like trametinib or cobimetinib) [1–5] and the use of antibodies against immune checkpoint inhibitors like CTLA-4 (ipilimumab) [6–9] or PD-1 (pembrolizumab and nivolumab) [10–13]

Treatment with immunotherapy achieves unprecedented long survival rates, with a 3-year survival rate of 20-40% [7]. Ipilimumab was the first approved immunotherapy drug based on an improvement in overall survival due to long term clinical benefit in a minority of patients [12]. In the case of BRAF mutant melanoma patients, treatment with BRAF/MEKi has also demonstrated improvements in survival [2, 3, 8]. BRAF/MEKi achieves a high response rate, with activity in nearly 80% of patients [2, 3, 8]. Despite these rapid and frequent responses, the benefits of BRAF/MEKi are usually transient, with a median disease-free survival of less than 12 months because of the almost universal development of acquired resistance [2, 6, 14]. Therefore, interest in combining both treatment modalities—MAPK pathway inhibition and immunotherapy—has grown, with the goal of achieving improved long-term survival rates [15–19].

It remains controversial as to which of these treatments should be used in first-line setting [20, 21] and whether combining them (either simultaneously or sequentially) could improve their activity [17, 19]. Preclinical data support the use of sequential immunotherapy in tumors responding to BRAF/MEKi rather than waiting until progression has occurred following BRAF/MEKi treatment [22, 23]. BRAF/MEKi can produce changes in the tumoral microenvironment of responding lesions, which can then favor a response to immunotherapy [17, 23]. An increase in tumor infiltration by CD8+ lymphocytes with a decrease in regulatory T cells (Tregs) and other immunosuppressive cells, as well as an increase in PD ligand (PD-L1) expression on tumor cells, have also been observed in tumors responding to BRAF/MEKi [5]. However, no clinical data are available that support the use of the sequential treatment in this setting.

What follows is a case report of fatal gastrointestinal (GI) toxicity in a melanoma patient who achieved a complete response (CR) with the combination of dabrafenib and trametinib followed by ipilimumab.

## CASE REPORT

**Figure 1:**
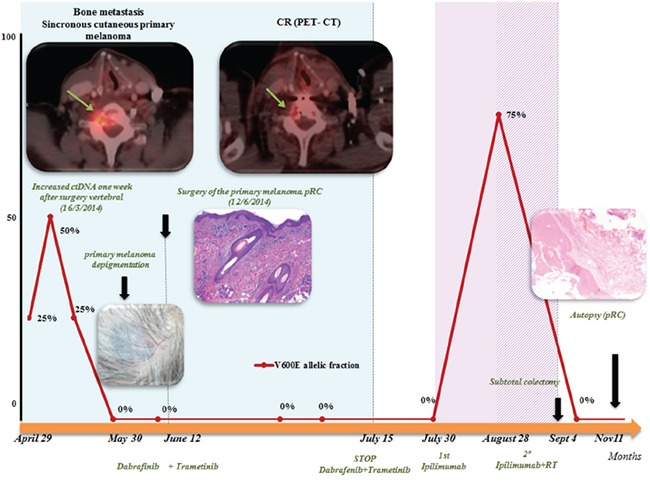
Summary of evolution: correlation of clinical data and BRAFV600E determination on serial ctDNA analysis: determination of BRAFV600 on ctDNA correlated well with clinical evolution A rapid negativization was observed 4 weeks after starting BRAF/MEKi. A peak after surgery of the bone metastases was observed, but it was transitory. After the first dose of ipilimumab, a transitory increase was also observed. Positron emission tomography (PET)-computed tomography (CT) demonstrated a complete response (CR) to dabrafenib and trametinib combination. Abbreviations: ctDNA: circulating tumoral DNA; pCR: pathological complete response;

**Figure 2:**
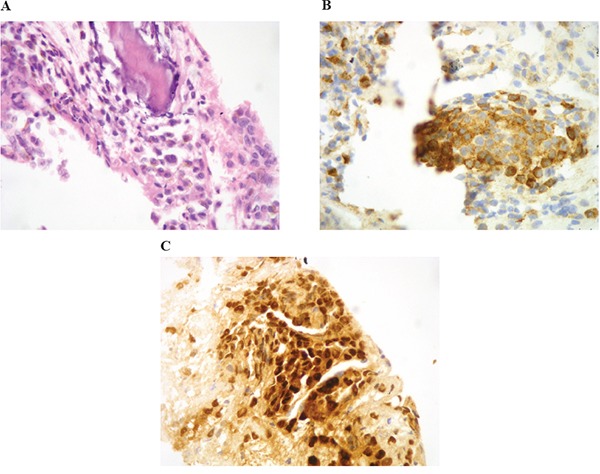
Core-biopsy of osteolithic lesion in the D9 vertebra showing bone infiltration by proliferation of round cells with hyperchromatic nucleus and occasional prominent nucleoli **A.** The immunohistochemical stains for HMB-45 **B.** and S-100 **C.** were positive.

**Figure 3:**
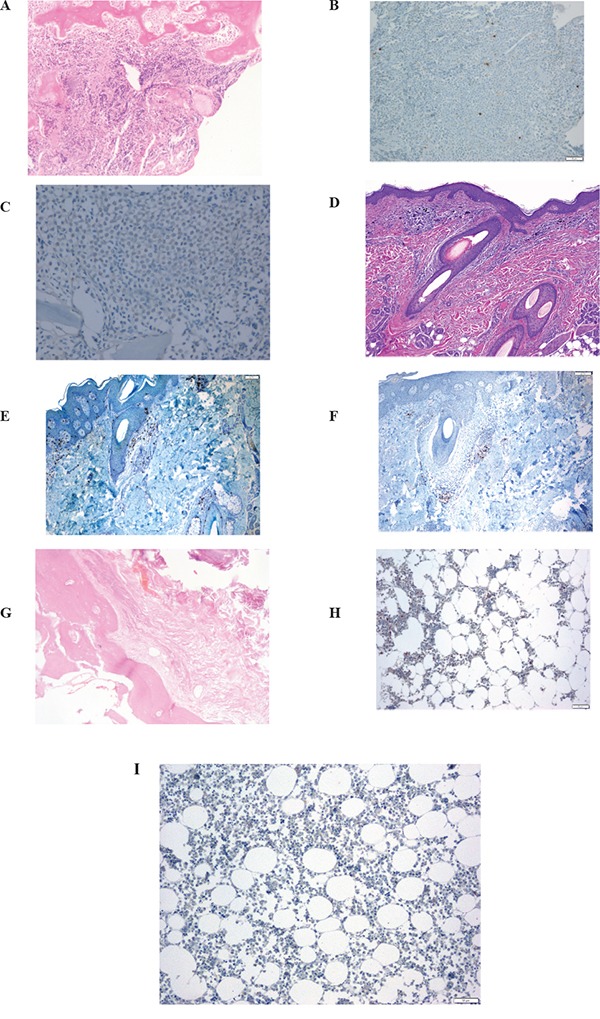
Serial tissue biopsies A, B, C: bone biopsy after two weeks on BRAF/MEKi treatment. **A.** Bone infiltration of C7 vertebra by a solid proliferation or round cells; **B.** Low CD8+ lymphocyte infiltration at bone metastases; **C.** negative programmed death-ligand (PD-L)1 immunohistochemistry at the bone lesion. D, E, F: primary cutaneous melanoma after six weeks on BRAF/MEKi treatment. **D.** pathological CR on primary cutaneous lesion, without fibrosis, after 6 weeks of treatment with BRAF/MEKi; **E.** intense CD8+ infiltration at the primary site; **F.** PD-L1 positivity on infiltrating lymphocytes at the primary tumor biopsy. G, H, I: bone biopsy from autopsy, after ipilimumab treatment. **G.** Body of the D9 vertebra with extensive fibrotic tissue with no residual tumor at autopsy; **H.** scanty lymphocytic infiltrate at bone (autopsy); **I.** PD-L1 negative at bone lesion (autopsy).

**Figure 4:**
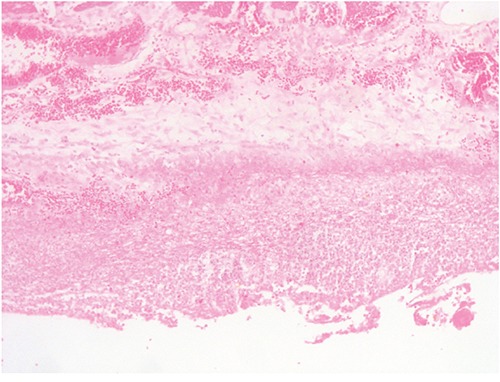
Subtotal colectomy Enterocolitis due to ipilimumab. Large bowel wall with necrosis and lymphocytic and leucocytic infiltration.

The patient was a 63-year-old man with no significant medical history. In November 2013, he visited the traumatology department owing to cervical pain. Magnetic resonance imaging (MRI) showed a lytic lesion at the C7 vertebrae with infiltration of both pedicles, raising suspicions of bone metastases. The PET-CT showed two hypermetabolic lesions, one at C7 (SUV 6.1) and another at D9 vertebrae (SUV 4.9), without visceral spread (Figure 1). On physical examination, a heterogeneous, hyperpigmented, three centimeter cutaneous lesion was found on the left parieto-occipital area of the scalp, consistent with primary melanoma. Core biopsy of the lesion at D9 vertebrae confirmed infiltration by melanoma cells, positive for both S-100 and HMB45 by immunohistochemistry (Figure 2). Routine blood tests showed no relevant data except high lactate dehydrogenase (LDH) levels. BRAFV600E mutation was detected in both tumoral tissue and circulating tumoral DNA (ctDNA) obtained from peripheral blood. In April 2014, the patient started treatment with dabrafenib (150 mg twice daily) in combination with trametinib (2 mg once daily), with rapid clinical improvement, depigmentation of the primary cutaneous lesion (Supplementary Figure 1), and negativization of the BRAFV600E mutation in ctDNA (Figure 1). In May 2014, after two weeks of treatment with BRAF/MEKi, a cervical vertebrectomy was performed to avoid neurological complications, followed by the surgical resection of the primary cutaneous lesion four weeks later. Surgical specimens confirmed melanoma infiltration at the bone lesion (Figure 3A and Supplementary Figure 2A-2B) with low CD8+ lymphocyte infiltration (Figure 3B) and negative PD-L1 immunohistochemistry (Figure 3C). Four weeks later, a complete melanoma regression without fibrosis at the primary site (Figure 3D) with an intense CD8+ infiltration (Figure 3E) and PD-L1-positive infiltrating lymphocytes (Figure 3F) was identified. In July 2014, a PET-CT showed a complete morpho-metabolic response (Figure 1). Treatment with dabrafenib and trametinib was stopped in July 2014 to perform radiotherapy of the bone lesions for consolidation therapy (30 Gy, 6 Gy/fraction). Treatment with ipilimumab 3 mg/kg intravenously (i.v.) and radiotherapy was started on August 2014. After the second dose of ipilimumab, the patient developed diarrhea, but he did not come to the hospital as he was instructed to do. After ten days of progressive deterioration with more frequent diarrhea and abdominal pain, the patient was finally admitted to the hospital. He presented with an acute abdomen with a perforated colon. Subtotal colectomy was performed, and pathological examination showed intestinal necrosis with increased lymphocyte infiltration in the wall, leading to a diagnosis of enterocolitis (Figure 4 and Supplementary Figure 3). The patient was admitted to the intensive care unit, and a high dose of corticosteroid treatment was administered. CT scan showed no metastatic lesions, while BRAFV600E mutation analysis in ctDNA showed negative results, even though there was a temporary peak of BRAFV600E in ctDNA four weeks after the last dose of ipilimumab. After eight weeks, the patient presented with a new colon perforation, even though corticosteroid treatment had been maintained. He and his family refused further treatment and opted for palliative care. Autopsy showed peritonitis with colon perforation and enterocolitis as the cause of the death (Supplementary Figure 4A); lung aspergilloma was also found (Supplementary Figure 4B). No melanoma metastases were found in the bones or at visceral sites, confirming a pathological CR (Figure 3G) with infiltration by CD8+ PD-L1-negative lymphocytes (Figure 3H and 3I).

## DISCUSSION

Among the immune-related adverse events (irAEs) caused by ipilimumab treatment, GI toxicity is the most frequent. Around 20-30% of patients treated with ipilimumab in clinical trials [8, 24] and in the clinical setting [25] present with GI toxicity. Early treatment of this complication has been shown to be very important in avoiding life-threatening complications, such as the bowel perforation that occurred in this patient [25]. In the first published phase III trial of ipilimumab in metastatic melanoma, five of 511 patients treated with ipilimumab died owing to bowel perforation [8]. However, in more recent studies, no fatal adverse events related to ipilimumab have been reported, with severe colitis or diarrhea in only 7.7% and 4.5% of patients, respectively [26]. This decrease in toxicity rate over time reflects a learning curve in the management of ipilimumab toxicity.

In our patient, the most probable cause of this fatal complication was the delay in communicating and treating GI symptoms. Other possible factors that could have increased the risk of complications were the concomitant treatment with radiotherapy or the previous treatment with the combination of BRA/MEKi.

A previous published case report described how radiotherapy in combination with ipilimumab shows marked activity via the so-called abscopal effect [27]. According to data from a phase I trial, radiotherapy increases cancer antigen presentation due to tumor necrosis and enhances the diversity of the T cell receptor (TCR) in intratumoral cells, without increasing toxicity when combined with ipilimumab [28].

Treatment with ipilimumab following BRAF inhibitors has been previously reported in patients progressing to the targeted inhibitor [21]. In this setting, no increase in toxicity was found [2]. The possibility that ipilimumab is more toxic in patients achieving response than in those progressing to BRAF inhibitors is unlikely, but clinical trials ongoing will clarify this issue. Here, ipilimumab treatment was initiated in a responding melanoma, instead of waiting until progression, because both the patient's preference for stopping oral drugs and trying a “definitive” treatment, and because radiotherapy requires interruption of BRAF/MEKi treatment in order to avoid toxicity.

The GI tract is a site of high immune system activity. The resident microbiota contain thousands of different bacteria that play an important role in lymphocyte education, with possible value as predictive markers of response [29] and toxicity to ipilimumab [30]. Lymphocytes and other immune cells on the intestinal wall (mucosal associated lymphoid tissue, MALT) are very complex and include different innate and adaptive immune cells, including the largest population of T cells in the body. Most of these lymphocytes are CD4+, but other subsets of lymphoid cells, such as TH17 cells, CD8+Tregs, natural killer (NK) cells, and others, are also present [31]. It is possible that lymphocytes infiltrating the bowel wall could be activated by ipilimumab in a non-standard manner after increasing their checkpoint inhibitor membrane presentation with a MEK inhibitor [32].

In the clinical setting, several phase I trials have reported toxicity related to the combination of BRAF/MEKi and ipilimumab. A trial testing the BRAF inhibitor vemurafenib plus ipilimumab was stopped owing to hepatotoxicity [33]. The combination of dabrafenib plus trametinib and ipilimumab produced colon perforation in two of the seven patients treated in the phase I trial, while no toxicity was observed when trametinib was not included in the combination [34].

The present case had a CR with sequential BRAF/MEKi followed by ipilimumab. CR by PET-CT was observed after dabrafenib plus trametinib, as well as negativization of BRAFV600E mutation in ctDNA. Achieving CR to dabrafenib and trametinib combination is unusual (less than 10% of patients achieve a CR) [1], although the low tumor burden of this particular case could be the reason for this extraordinary response.

Although pathological CR assessed in the autopsy could be due exclusively to the dabrafenib and trametinib combination, and not necessarily to sequential treatment with ipilimumab, preclinical data suggest that immunotherapy could be more active in patients responding to BRAF/MEKi, instead of delaying treatment until MAPK inhibitor resistance occurs [5]. Following a response to MAPK pathway inhibition, there is an increase in PD-L1 expression on tumor cells and a higher lymphocytic infiltration (CD8/CD4), as well as, a decrease in Treg infiltration and immunosuppressive cytokines (IL6, IL10, VEGF) [5, 35]. In addition, melanosomal antigen expression is higher following increased microphthalmia-associated transcription factor MITF levels owing to blockade of the MAPK pathway. Conversely, when tumors progress to BRAF inhibitors, MITF is downregulated and melanosomal antigen expression is decreased. Due to the paradoxical activation of the MAPK pathway in T cells by BRAF inhibitor, functionality is increased in T cells. Although there are reports of higher expression of PD-L1 in human biopsies after progression to BRAF inhibitors [16], this is not universally observed, and preclinical data in cell lines have demonstrated that its occurrence depends on the resistance mechanism [36]. Given that resistance mechanisms are heterogeneous intra-patient and intra-lesion, PD-L1 expression in tissues after progression to BRAF inhibitors will be variable and weaker than in responding lesions. In our patient, after six weeks of treatment with BRAF/MEKi, an increase in CD8+ PD-L1+ lymphocyte infiltration was found at the cutaneous primary melanoma in complete regression (Figure 3D-3F), whereas there was no infiltration by CD8+ cells on bone metastases biopsy performed two weeks after starting BRAF/MEKi (Figure 3A-3C). After ipilimumab treatment, a moderate CD8+ PD-L1-lymphocyte infiltration was found at bone metastases with no residual tumor infiltration (Figure 3G-3I). It is possible, however, that the immunohistochemistry analysis for PD-L1 yielded false negative results because of the decalcification of the tissue sample (Figure 3I).

Recent data demonstrated that long-term survival is 20% after dabrafenib and trametinib combination treatment (progression-free survival [PFS] at three years of 21%). Survival is especially high in patients with normal pretreatment LDH levels or patients who achieve CR, with an overall survival (OS) in this subset at three years of 62% [2]. It could be possible to treat successfully those patients who achieve a CR to dabrafenib and trametinib without any additional combinations. Clinical and molecular predictive factors that help to select the best treatment options for every case are urgently needed.

We have developed a reverse transcription polymerase chain reaction (RT-PCR) based assay to analyze and quantify BRAFV600E mutation in plasma and serum with a sensitivity level of 0.005% mutation load. In our experience, about 70% of BRAF metastatic melanoma patients have a detectable mutation on pretreatment ctDNA, and those patients with detectable BRAFV600E mutation on ctDNA pretreatment have a poor outcome, with a median OS of 7 months versus 22 months for those without pretreatment BRAFV600E mutation on ctDNA [37]. In addition, we have observed that 60% of patients with a BRAF mutation in pretreatment plasma/serum samples achieved BRAF ctDNA negativization during treatment [38]. Poor outcomes have been observed in those patients with persistence of BRAF mutation in blood after the first weeks on treatment, with rapid disease progression leading to death within a few months [38].

In the present case, the BRAFV600E mutation was detected in the pretreatment plasma sample, in spite of the low tumor burden. A rapid decrease was observed after dabrafenib and trametinib combination, with a complete negativization achieved in four weeks. After surgery of the metastatic bone lesion, a temporary increase in BRAFV600E ctDNA was found. It is well known that this phenomenon is due to the release of DNA into the blood after trauma or surgical procedures. A transitory peak of BRAFV600E ctDNA after ipilimumab treatment was observed, and although a false positive result cannot be ruled out, this finding suggests a transitory progression of the disease with a final response to ipilimumab, as response to immunotherapy needs more time to activate the immune response. Our group has demonstrated that monitoring disease evolution by BRAF ctDNA analysis during treatment has a good correlation with clinical evolution and could help us assess response to therapy before it is detected by conventional imaging techniques [38].

## CONCLUSIONS

Ipilimumab-associated fatal toxicities are observed mainly in cases wherein management of toxicity is delayed, as in the present case.

Combination modalities with sequential or concomitant BRAF/MEKi and ipilimumab must be carefully evaluated in the context of clinical trials, given that several cases of colon perforation have been reported with the combination of MEK inhibitors and ipilimumab [34].

It is known that some prognostic factors, such as normal LDH levels or CR, predict a good evolution with simple treatments like BRAF/MEK inhibitor combination [1]. Identifying prognostic and predictive factors will help select those patients who need more aggressive treatments and those who can achieve long-term survival using less complex treatments.

## SUPPLEMENTARY MATERIALS FIGURES


