# Supplementary material for: Fatal gastrointestinal toxicity with ipilimumab after BRAF/MEK inhibitor combination in a melanoma patient achieving pathological complete response
Source: Oncotarget. 2016 Jul 18;7(35):56619–27. doi: 10.18632/oncotarget.10651 (PMC5302939; doi:10.18632/oncotarget.10651)
Supplement: Supplementary file 1 [file oncotarget-07-56619-s001.pdf]

Fatal gastrointestinal toxicity with ipilimumab after BRAF/MEK inhibitor combination in a melanoma patient achieving pathological complete response

SUPPLEMENTARY FIGURES

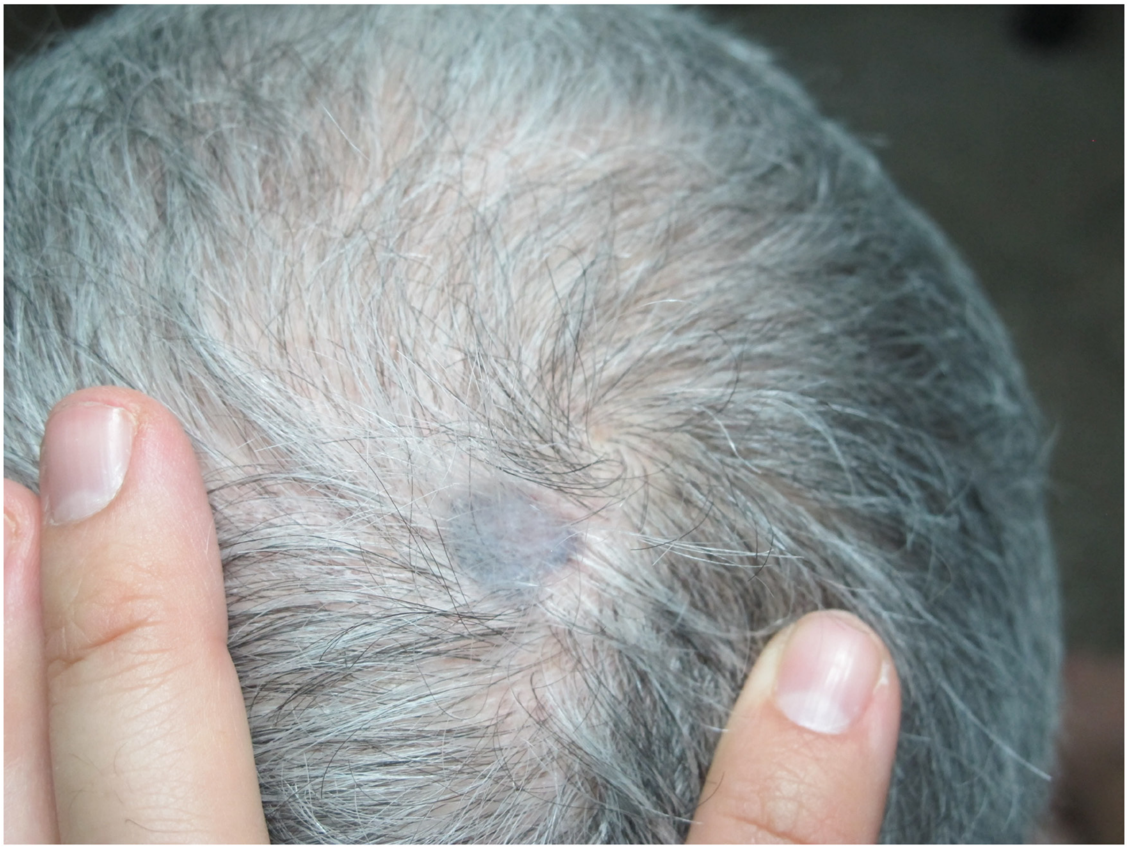

Supplementary Figure S1: Primary cutaneous lesion: depigmented lesion after one month on BRAF/MEKi.

A

B

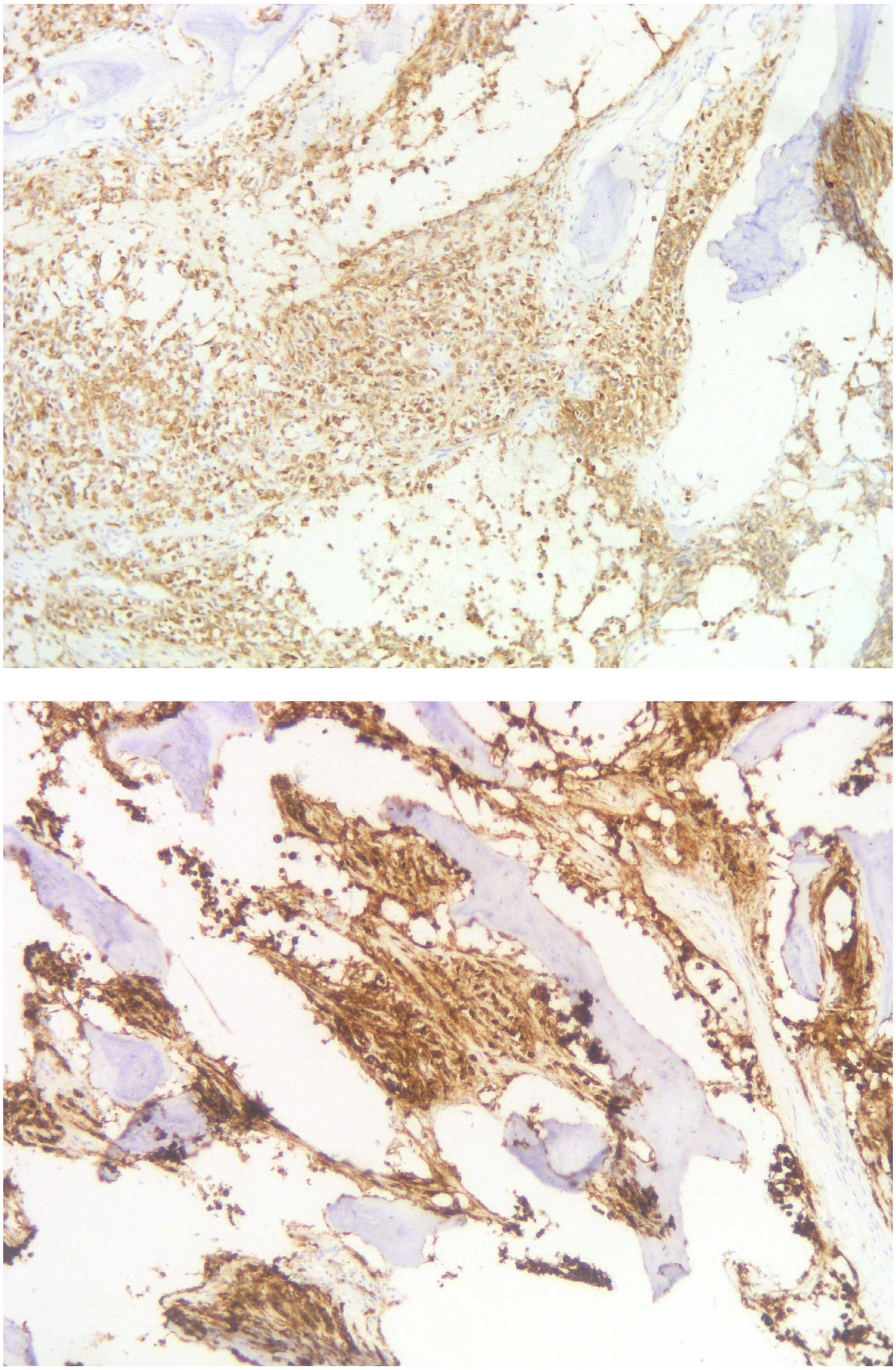

Supplementary Figure S2: Melanoma bone metastases: immunohistochemical stains for HMB-45 A. and Melan A **B.**

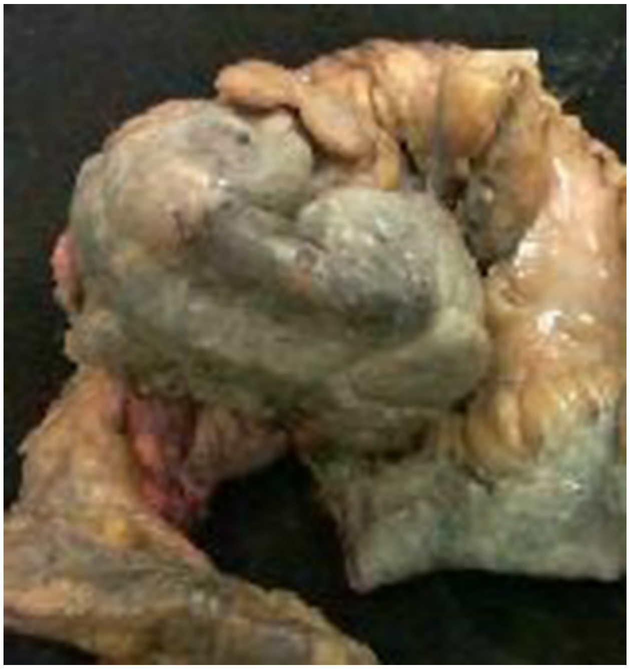

Supplementary Figure S3: Gross specimen of subtotal colectomy with necrosis and hemorrhage.

A

B

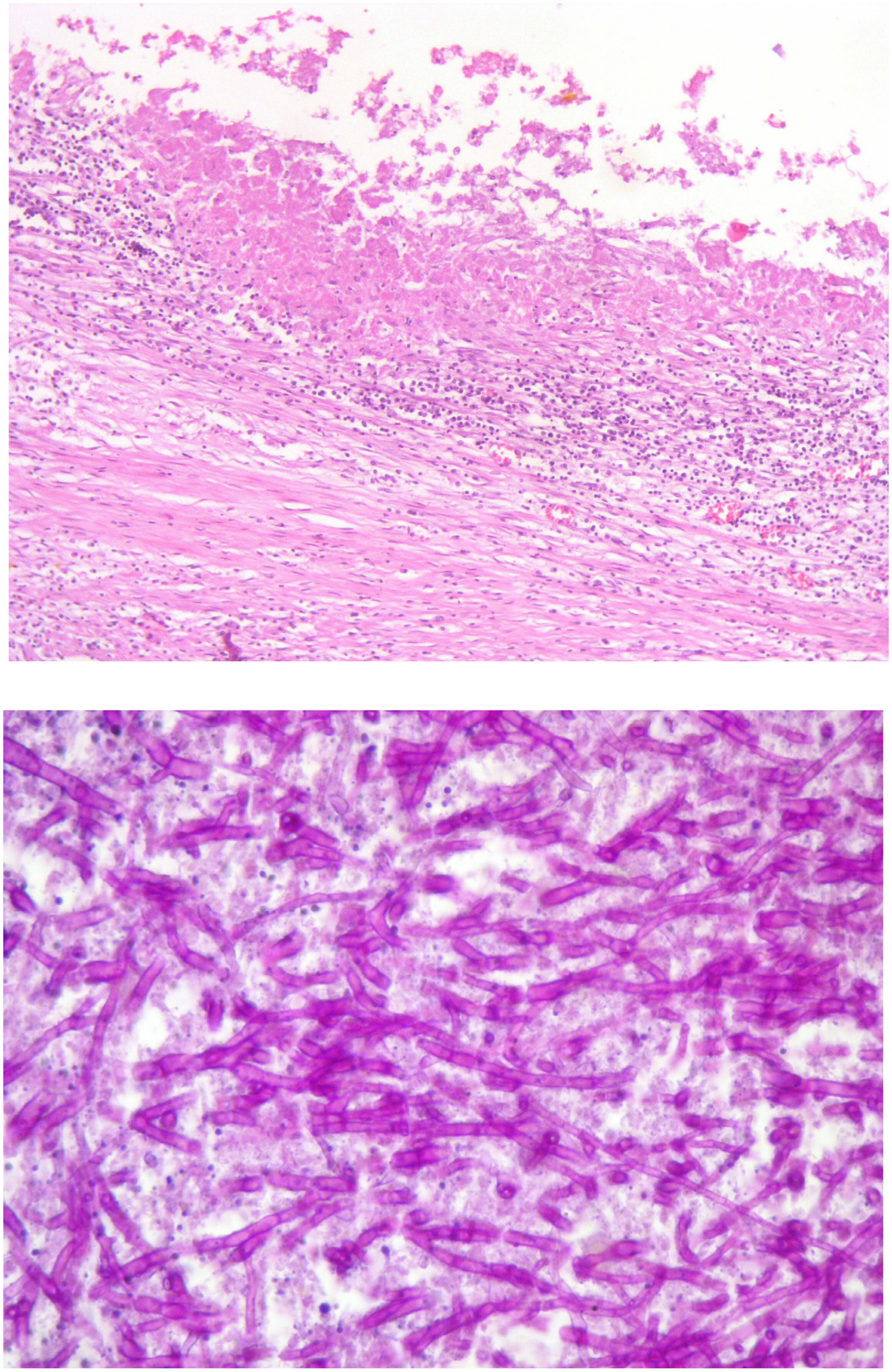

Supplementary Figure S4: Autopsy: Peritoneal surface with extensive leucocytic infiltration A.; Lung parenchyma showing a prominent invasion by septated and ramified fungal hyphae consistent with Aspergillus **B.**
